# Statin Use Associates With Risk of Type 2 Diabetes via Epigenetic Patterns at *ABCG1*

**DOI:** 10.3389/fgene.2020.00622

**Published:** 2020-06-16

**Authors:** Yuwei Liu, Yu Shen, Tao Guo, Laurence D. Parnell, Kenneth E. Westerman, Caren E. Smith, Jose M. Ordovas, Chao-Qiang Lai

**Affiliations:** ^1^School of Public Health, Fudan University, Shanghai, China; ^2^Nutrition and Genomics Laboratory, JM-USDA Human Nutrition Research Center on Aging at Tufts University, Boston, MA, United States; ^3^Department of Cardiology, Zhongnan Hospital of Wuhan University, Wuhan, China; ^4^USDA Agricultural Research Service, Nutrition and Genomics Laboratory, JM-USDA Human Nutrition Research Center on Aging at Tufts University, Boston, MA, United States; ^5^IMDEA Food Institute, CEI UAM + CSIC, Madrid, Spain; ^6^Centro Nacional de Investigaciones Cardiovasculares (CNIC), Madrid, Spain

**Keywords:** statin, *ABCG1*, methylation, type 2 diabetes, cg06500161

## Abstract

Statin is the medication most widely prescribed to reduce plasma cholesterol levels. Yet, how the medication contributes to diabetes risk and impaired glucose metabolism is not clear. This study aims to examine the epigenetic mechanisms of *ABCG1* through which statin use associates with risk of type 2 diabetes. We determined the association between the statin use, DNA methylation at *ABCG1* and type 2 diabetes/glycemic traits in the Framingham Heart Study Offspring (FHS, *n* = 2741), with validation in the Women’s Health Initiative Study (WHI, *n* = 2020). The causal effect of statin use on the risk of type 2 diabetes was examined using a two-step Mendelian randomization approach. Next, based on transcriptome analysis, we determined the links between the medication-associated epigenetic status of *ABCG1* and biological pathways on the pathogenesis of type 2 diabetes. Our results showed that DNA methylation levels at cg06500161 of *ABCG1* were positively associated with the use of statin, type 2 diabetes and related traits (fasting glucose and insulin) in FHS and WHI. Two-step Mendelian randomization suggested a causal effect of statin use on type 2 diabetes and related traits through epigenetic mechanisms, specifically, DNA methylation at cg06500161. Our results highlighted that gene expression of *ABCG1, ABCA1* and *ACSL3*, involved in both cholesterol metabolism and glycemic pathways, was inversely associated with statin use, CpG methylation, and diabetic signatures. We concluded that DNA methylation site cg06500161 at *ABCG1* is a mediator of the association between statins and risk of type 2 diabetes.

## Introduction

Dyslipidemia is a major risk factor for cardiovascular disease (CVD), which remains the leading cause of mortality worldwide. Lipid-lowering drugs, like statins, are the medication most widely prescribed to reduce plasma cholesterol levels (Chou et al., 2016; Zhan et al., 2018). With common and long-term use of such medications, a number of adverse effects have been reported. Specifically, statins have been associated with liver damage (Aguirre et al., 2013), muscle discomfort (Pirillo and Catapano, 2015) and diabetes (Kim D. W. et al., 2019; Kim Y. S. et al., 2019). Yet, how the medication contributes to diabetes risk and impaired glucose metabolism is not clear.

Type 2 diabetes is a complex disease that results from genetic and environmental factors and their interactions. Environmental factors, including diet, lifestyle and medications, may modify epigenetic status affecting the risk of type 2 diabetes (Vickers, 2014; Nilsson and Ling, 2017). Epigenome-wide association studies in several cohorts have shown strong associations between DNA methylation at CpG sites in ATP binding cassette subfamily G member 1 (*ABCG1*) and risk of type 2 diabetes (Hidalgo et al., 2014; Dayeh et al., 2016; Davegardh et al., 2018). ABCG1, a member of the ATP-binding cassette (ABC) protein family, functions in removing excess cholesterol from peripheral tissues and transporting it to the liver (Matsuo, 2010). *ABCG1* expression has been reported to be regulated by statins in Caco-2 cells and COPD patients (Genvigir et al., 2011; Obeidat et al., 2015). Thus, we hypothesized statin use induces epigenetic changes that affect gene expression to increase the risk of type 2 diabetes.

This study aims to characterize the association between the use of statins, DNA methylation at *ABCG1* and glycemic traits. For this purpose, we used the Framingham Heart Study Offspring (FHS) as the discovery population and validated the findings in the Women’s Health Initiative Study (WHI). We then determined if statin use had causal effects on the risk of type 2 diabetes using a two-step Mendelian randomization approach (Relton and Davey Smith, 2012; Caramaschi et al., 2017; Zhu et al., 2018). Furthermore, we determined whether altered DNA methylation at CpGs contributed to specific metabolic pathways on the pathogenesis of type 2 diabetes.

## Materials and Methods

### Framingham Heart Study (FHS)

The FHS follows several community dwelling generations of participants recruited in Framingham, MA, since 1948 (Dawber et al., 1951). In 1971, 5124 offspring, self-identified as European ancestry, from the original cohort and spouses were recruited (Kannel et al., 1979). In exam 8 (2005–2008) of this offspring cohort, participants completed a comprehensive set of dietary and health assessment questionnaires. The use of statins was obtained from the questionnaires. Statin users were defined as participants who took any type of statins, whereas non-statin users were those who did not use statin, but might or might not have taken other lipid-lowering drugs. LDL-C was estimated using Friedewald equation (Lindsey et al., 2004). DNA methylome and transcriptome analyses were performed on blood samples collected from 2741 participants (Marioni et al., 2015). These data were obtained from dbGaP (accession: phs000007.v29.p10; downloaded on September 27, 2017).

### Women’s Health Initiative Study (WHI)

Starting from 1993, the WHI study recruited over 160,000 women into a long-term national health study that focuses on strategies for preventing heart disease, breast and colorectal cancer, and osteoporosis in postmenopausal women The Women’s Health Initiative Study Group (1998). Participants (*n* = 2020) from a combined case-control and pseudo-case-cohort were included in this study. Blood samples used for measurement of DNA methylation and clinical biochemistry were taken at Exam 1. Data was requested from dbGaP (accession: phs000200.v11.p3; downloaded on September 27, 2017). At Exam 1, participants also completed a comprehensive set of dietary and health assessment questionnaires. The use of statins was obtained from the questionnaires. The same definition of statin users and non-statin users in FHS was applied to WHI.

### DNA Methylation Analysis

Raw IDAT files of DNA methylation analysis were quality-controlled and processed as described (Hidalgo et al., 2014; Marioni et al., 2015; Lai et al., 2018). Briefly, we used a β score to measure methylation signal as the proportion of the total methylation-specific signal, and the detection *P*-value as the probability that the total intensity for a given probe fell within the background signal intensity. Then, we excluded any CpG probes with a detection *P*-value > 0.01 and missing sample percentage > 1.5%, or > 10% of samples lacking sufficient intensity. We performed normalization on the filtered β scores using the ComBat package in R (Morris et al., 2014). Principal components were calculated on the β scores of samples that passed quality control using the prcomp function in R.

### Genotyping

Genotypes for 2176 individuals from FHS were requested from dbGaP (accession: phs000342.v18.p11) as part of the NHLBI SNP Health Association Resource (SHARe) project, with initial QC and imputation having been performed previously. Briefly, approximately 500,000 SNPs were genotyped on the Affymetrix500k and Affymetrix50k chips and filtered for Hardy-Weinberg equilibrium, call rate, and minor allele frequency. These genotypes were then phased using MACH and imputed to the November 2010 release of 1000 Genomes using Minimac. Additional filters were applied to the imputed dosage data as follows: only samples associated with subjects who also had methylation data available were retained, and SNPs were filtered for an imputation quality score (R^2^) > 0.9. Dosages were converted to hard-calls if the dosage was within 0.2 of an integer allele count (0/1/2) and otherwise were set as missing.

Genotypes for 1966 individuals from WHI were available from dbGaP (accession: phs000746.v2.p3) as part of an imputation and harmonization effort across six GWAS sub-studies. Quality control and preprocessing steps were applied in each of the sub-studies prior to imputation (details available at dbGaP). Genotypes were then phased and aligned to the 1000 Genomes reference panel using BEAGLE and Minimac. The imputed dosages of 5,298,674 SNPs were retrieved from dbGaP and filtered for imputation quality score > 0.3, converted to hard-calls within 0.1 of an integer allele count (otherwise set as missing), and merged across sub-studies.

For both genotyped cohorts, SNP IDs, loci, and allelic information were annotated using the 1000 Genomes Phase 3 download from dbSNP (download date: April 13, 2018). Genotype processing was performed using PLINK 1.9 and 2.0 [URL^1^; (Chang et al., 2015)].

### Transcriptome Analysis

We obtained FHS transcriptome data from dbGaP under accession phe00002.v6. Transcriptome analysis was conducted in exam 8 using the Affymetrix Human Exon 1.0 ST array for 17873 probes with mRNA from whole blood samples collected from 1616 participants from the Offspring Cohort after overnight fasting. Detailed descriptions of transcriptome analysis procedures are available (Katz et al., 2006).

### Statistical Methods

#### Associations Between Statin Use and DNA Methylation of ABCG1

Focusing on *ABCG1*, we investigated all 31 CpG sites (cg00177237, cg00222799, cg01176028, cg01289965, cg01881899, cg02241241, cg02316713, cg02370100, cg02473680, cg05046272, cg0544165, cg05639842, cg06030219, cg06500161, cg07397296, cg07875759, cg08663969, cg08841829, cg10192877, cg11662315, cg14982472, cg16068063, cg17526396, cg20214535, cg21410080, cg23245768, cg25615529, cg26519745, cg26767954, cg26768067, cg27243685) that are in or near the *ABCG1* gene. In the discovery stage in FHS, we modeled the association between statin use and methylation score at these *ABCG1* CpG sites in (i) all participants and (ii) the participants who were not using antidiabetic medications using a generalized linear model, adjusting for family relationship, sex, age, smoking, alcohol and cell-type heterogeneity (basic model). Moreover, we used an LDL-C and TG adjusted model in which low density lipoprotein cholesterol (LDL-C) and triglycerides (TG) were added to the basic model. The analysis was implemented by the log link function in a GENMOD Procedure in SAS studio (University Edition). We fitted the identical model to the replication samples in WHI using the statistically significant CpG sites identified in FHS (*P*-value cut-off was set as 1.6E-3, Bonferroni correction), where adjustment for family relationship and gender were not needed, as WHI is an un-related female cohort.

#### Correlations Between DNA Methylation of ABCG1 and Diabetic Traits

In the discovery stage, we examined the association between methylation of *ABCG1* CpG that were associated with statin use (*P-*value < 1.6E-3 in basic model) in both cohorts for diabetes-related traits. A generalized linear model was implemented with methylation measures as dependent variables and type 2 diabetes (selection criteria: fasting glucose ≥ 126 mg/dL, or use of diabetic medication) as the predictor using logit link function in a GENMOD Procedure in SAS, adjusting for family relationship, sex, age, smoking, alcohol use and cell-type heterogeneity. Participants without diabetes were included for a similar association study in the Log-Linear Model of GENMOD Procedure with methylation measures as dependent variables, while log-transformed glucose or insulin levels were used as predictors, adjusting for family relationship, sex, age, smoking, alcohol use and cell-type heterogeneity. We fitted the identical models in the replication samples in the WHI study. As noted previously, family relationship and gender were not adjusted in WHI because it is an un-related female cohort.

#### Two-Step Mendelian Randomization (MR) Approach

We performed a two-step MR approach to test the causal role of statin use on diabetic traits (fasting glucose and insulin) in the non-diabetic population and type 2 diabetes in the whole population. The first step MR analysis was implemented in the FHS study with the second in the WHI cohort using GSMR (Generalized Summary-data-based on Mendelian randomization) in GCTA (Zhu et al., 2018). In the first step, genome-wide SNPs were first tested for association with the statin use (exposure) and cg06500161 methylation (outcome) using a mixed linear model association (GCTA-MLMA), adjusting for sex, age, smoking, alcohol use, cell-type heterogeneity, and family relationship (Yang et al., 2014). These tests were repeated in the LDL-C and TG adjusted models. Then, the summary data from MLMA were used to estimate the causal effect of statin use on cg06500161 using GSMR. We selected the genome-wide significant SNPs (*P-*value < 5E-5) associated with the exposures as the instrumental variable in the basic and LDL-C and TG adjusted models, respectively. We used the HEIDI-outlier approach to remove SNPs that have effects on both the exposures and the outcomes. The remaining SNPs were then tested for the association with the outcomes for causal effect.

In the second step, SNPs were firstly tested in the identical models for association with cg06500161 methylation (exposure) as well as diabetes, log-transformed fasting glucose and insulin levels (outcomes), separately. In this step, the sex, age, smoking, and alcohol use, were adjusted. Next, the summary data from MLMA were utilized to calculate the causal effect of cg06500161 on type 2 diabetes and related traits using GSMR. We selected the genome-wide significant SNPs (*P-*value < 5E-5) associated with the exposures as the instrumental variable in diabetes, glucose and insulin models, respectively. We also used the HEIDI-outlier approach to remove SNPs that have effects on both the exposures and the outcomes. The remaining SNPs were then tested for the association with the outcomes for causal effect. Then the causal effects of statins on type 2 diabetes and related phenotypes through cg06500161 methylation were calculated by multiplying the causal effect of statin use on cg06500161 from Step 1 and the causal effect of cg06500161 on the outcomes (type 2 diabetes and related traits) from Step 2.

#### Gene Sets Analysis

To uncover the connection among statins, epigenomic modification and transcriptional network, we conducted gene set enrichment analysis (GSEA) in light of the identified methylation sites in the selected non-diabetic participants of the FHS study. Firstly, we performed separate association analyses between all transcripts and statins, cg06500161 methylation, log-transformed glucose as well as insulin levels using a default mixed linear model in OSCA (OmicS-data-based Complex Trait Analysis, version 0.42), adjusting for sex, age, smoking, alcohol, and cell-type heterogeneity in the LDL-C and TG adjusted model (Zhang et al., 2018). The CpG-associated (*P*-value < 0.05) genes were then evaluated for overlaps with those genes correlating (at *P*-value < 0.05) with statins, glucose or insulin levels. The number of overlapped genes was counted within each pathway. *P*-values and FDR q values were calculated for both KEGG and Reactome pathway data using GSEA with 45956 gene entries.

## Results

### Statin Use Is Associated With ABCG1 CpG Methylation

Clinical characteristics for all participants in both FHS and WHI cohorts are listed according to statin-use status and T2D in Table 1. Using an independent *t*-test or Fisher’s exact test, we examined the differences between statin and non-statin groups, excluding participants taking antidiabetic medication, and between T2D and non-T2D groups in both populations. In both cohorts (Table 1), participants who took statins or those with T2D, tended to have an increased risk for abnormal lipid profile, except for cholesterol (total cholesterol and LDL-C), compared to non-statin users or non-T2D participants (Table 1). One important observation was the percentage of T2D patients who took statin was about twice that of non-T2D participants in both FHS (56% vs 28%) and WHI (21% vs 10%). This suggests that T2D risk is strongly associated with statin use.

**TABLE 1:** Clinical characteristics of participants from the FHS and WHI studies.

	*A*^1^		*B*^2^	
	Statin^3^	Non-Statin	T2D^4^	Non-T2D
**FHS**				
N	654	1287	318	1858
Age, years	68.0 ± 8.2	64.6 ± 9.0*	68.7 ± 8.4	65.7 ± 8.9*
Female (%)	319(49)	766(60)*	135(43)	1055(57)*
Statin user (%)	654(100)	0*	179(56)	514(28)*
Smoker (%)	45(7)	119(9)	21(6.6)	161(9)
Drinker (%)	553(85)	1062(83)	223(70)	1554(84)*
TC (mg/dL)	167.7 ± 30.7	198.9 ± 33.8*	165.9 ± 35.1	189.0 ± 36.0*
HDL-C (mg/dL)	54.4 ± 15.0	60.7 ± 19.0*	48.8 ± 16.6	59.0 ± 17.9*
LDL-C (mg/dL)	89.6 ± 25.3	115.9 ± 29.0*	87.5 ± 28.7	107.4 ± 30.5*
TG (mg/dL)	118.5 ± 61.3	111.6 ± 63.1*	146.2 ± 91.4	112.4 ± 69.2*
Glucose (mg/dL)	103.7 ± 11.9	101.0 ± 13.4*	139.3 ± 39.1	100.4 ± 9.3*
Insulin (pmol/L)	83.2 ± 58.6	65.7 ± 43.5*	111.0 ± 78.3	69.8 ± 49.0*
**WHI**				
N	190	1339	258	1762
Age (years)	66.6 ± 6.2	64.7 ± 7.1*	63.9 ± 6.7	64.5 ± 7.1
Female (%)	190(100)	1339(100)	258(100)	1762(100)
Statin user (%)	190 (100)	0*	53(21)	182(10)*
Smoker (%)	88(47)	627(48)	111(44)	811(47)
Drinker (%)	54(28)	381(29)	20(8)	511(29)*
TC (mg/dL)	249.6 ± 44.2	231.9 ± 42.2*	231.5 ± 46.2	234.0 ± 42.7
HDL-C (mg/dL)	50.7 ± 12.1	52.3 ± 12.7*	46.8 ± 11.9	52.4 ± 12.8*
LDL-C (mg/dL)	164.9 ± 41.4	151.1 ± 38.2*	148.7 ± 42.8	152.8 ± 38.9
TG (mg/dL)	174.8 ± 90.3	139.9 ± 73.5*	189.4 ± 133.5	141.2 ± 76.4*
Glucose (mg/dL)	100.1 ± 25.6	101.0 ± 29.0	173.7 ± 66.6	99.4 ± 26.0*
Insulin (pmol/L)	61.5 ± 38.7	60.5 ± 50.0	144.1 ± 330.9	59.2 ± 48.2*

^1^Participants who are not using antidiabetic medicines. ^2^All participants. ^3^Statin: Participants who are using statins. ^4^T2D: Participants who have type 2 diabetes. * Statistically significantly different between statin and non-statin groups, or between T2D and non-T2D groups in both FHS and WHI using independent *t*-test or Fisher’s exact test, *P* < 0.05.

To examine the association of statins with *ABCG1* methylation, we examined DNA methylation of *ABCG1* on the Infinium 450K array using the FHS cohort as a discovery set, with the WHI cohort used for validation. Table 2 lists CpGs that were associated with statin use in all participants of FHS, adjusting for age, sex, smoking, alcohol use, cell-type heterogeneity, and family relationship. After filtering out highly correlated CpG sites based on pairwise Pearson correlation tests (*r* ≥ 0.5), five CpGs were significantly associated with statin use, with cg06500161 (β = 0.0320, *P-*value = 1.00E-32) being the most significant. After additionally adjusting for serum low density lipoprotein cholesterol (LDL-C) and total triglycerides (TG), the association remained significant for cg06500161 (β = 0.0252, *P-* value = 1.55E-15). We then sought replication in the WHI cohort, observing that only cg06500161 (β = 0.0143, *P-*value = 3.15E-3) and cg05639842 (β = 0.0484, *P-*value = 6.15E-3) were significantly associated with statin use (Table 2). Thus, both CpGs remained statistically significant in the LDL-C and TG adjusted model in both FHS and WHI (Table 2). We found a similar association of statin use with *ABCG1* methylation in participants who were not using antidiabetic medication in both FHS and WHI cohorts (Table 3).

**TABLE 2:** *ABCG1* CpG sites associated with statin use in all participants from the FHS and WHI studies.

		FHS (Discovery)				WHI (Validation)			
CpG	Chr: Position^†^	*N*	B*	SE	*P-*value*	*N*	B*	SE	*P-*value*
***Basic model***									
cg06500161	21:42236477	2157	0.0320	3.02E-3	1.00E-32	1702	0.0143	4.83E-3	3.15E-3
cg01881899	21:42232595	2157	0.0749	0.0165	5.93E-6	1702	0.0138	0.0261	0.596
cg05639842	21:42219330	2157	0.0693	0.0196	3.98E-4	1702	0.0484	0.0177	6.15E-3
cg01176028	21:42233124	2157	0.0283	6.05E-3	2.88E-6	1702	6.85E-3	0.0167	0.682
cg02370100	21:42235147	2157	0.0373	0.0108	5.92E-4	1702	0.0257	0.0149	0.0846
***LDL-C and TG adjusted model***									
cg06500161	21:42236477	2153	0.0252	3.16E-3	1.55E-15	1564	0.0134	5.10E-3	8.36E-3
cg01881899	21:42232595	2153	0.0502	0.0173	3.76E-3	1564	0.0108	0.0265	0.684
cg05639842	21:42219330	2153	0.0582	0.0220	8.16E-3	1564	0.0470	0.0187	0.0121
cg01176028	21:42233124	2153	0.0183	6.86E-3	7.82E-3	1564	-0.0134	0.0173	0.437
cg02370100	21:42235147	2153	0.0344	0.0119	3.72E-3	1564	0.0215	0.0160	0.178

^†^The physical positions of CpG sites are based on GRCh38.p12. *Adjusted for age, sex (for FHS), smoking, alcohol use, cell-type heterogeneity, and family relationship (for FHS).

**TABLE 3:** *ABCG1* CpG sites associated with statin use in participants who are not using antidiabetic medication from the FHS and WHI studies.

		WHI (Validation)				FHS (Discovery)			
CpG	Chr: Position^†^	*N*	β	SE	*P-*value	*N*	β	SE	*P-*value
***Basic model***									
cg06500161	21:42236477	1923	0.0317	3.25E-3	1.00E-32	1502	0.0114	5.34E-3	0.0325
cg01881899	21:42232595	1923	0.0738	0.0187	8.08E-5	1502	4.78E-5	2.97E-2	0.999
cg05639842	21:42219330	1923	0.0765	0.0212	3.00E-4	1502	0.0621	0.0191	1.13E-3
cg01176028	21:42233124	1923	0.0273	6.73E-3	2.07E-5	1502	0.0192	0.0188	0.306
cg02370100	21:42235147	1923	0.0392	0.0115	6.51E-4	1502	0.0275	0.0165	0.0957
***LDL-C and TG adjusted model***									
cg06500161	21:42236477	1922	0.0257	3.29E-3	6.22E-15	1390	8.53E-3	5.54E-3	0.123
cg01881899	21:42232595	1922	0.0453	0.0204	0.0261	1390	-7.06E-4	0.0299	0.813
cg05639842	21:42219330	1922	0.0664	0.0236	4.97E-3	1390	0.0630	0.0202	1.85E-3
cg01176028	21:42233124	1922	0.0206	7.15E-3	3.97E-3	1390	-0.0241	0.0194	0.214
cg02370100	21:42235147	1922	0.0345	0.0124	5.52E-3	1390	0.0209	0.0177	0.238

*^†^The physical positions of CpG sites are based on GRCh38.p12.*

### *ABCG1* CpG Methylation Is Associated With Type 2 Diabetes and Related Traits

Because epigenetic analysis of other cohorts identified associations between DNA methylation at *ABCG1* and type 2 diabetes phenotypes, we tested both cg06500161 and cg05639842 for association with type 2 diabetes in the FHS cohort. Replication was sought in the WHI cohort. We observed greater type 2 diabetes prevalence with higher DNA methylation levels at cg06500161 (β = 12.4, *P*-value = 1.33E-10) in FHS (Table 4). Similar results with cg06500161 were found in WHI (β = 11.7, *P*-value = 4.70E-11). However, cg05639842 was not associated with type 2 diabetes in either FHS or WHI (Table 4).

**TABLE 4:** *ABCG1* CpG sites associated with type 2 diabetes, fasting glucose and fasting insulin in participants from the FHS and WHI studies.

			FHS (Discovery)				WHI (Validation)			
	CpG	Chr: Position^†^	*N*	β	SE	*P-*value	*N*	β	SE	*P-*value
***All participants***										
Type 2 diabetes	cg06500161	21:42236477	2176	12.4	1.93	1.33E-10	2020	11.7	1.77	4.70E-11
	cg05639842	21:42219330	2176	1.69	3.71	0.648	2020	16.0	11.73	0.172
***Non-diabetic participants***										
Blood glucose	cg06500161	21:42236477	1858	0.504	0.0946	1.02E-7	1762	0.853	0.174	9.22E-7
	cg05639842	21:42219330	1858	0.117	0.169	0.489	1762	−1.30	1.22	0.287
Blood insulin	cg06500161	21:42236477	1858	4.85	0.594	4.44E-16	1762	4.05	0.429	3.86E-21
	cg05639842	21:42219330	1858	1.42	1.20	0.0760	1762	0.842	3.21	0.793

*^†^The physical positions of CpG sites are based on GRCh38.p12.*

For the prediction of type 2 diabetes, we investigated DNA methylation at cg06500161 and cg05639842 with common risk factors for type 2 diabetes (fasting blood glucose and insulin levels) in non-diabetic participants in both cohorts. DNA methylation at cg06500161 was positively correlated with fasting glucose (β = 0.504, *P*-value = 1.02E-7) and insulin levels (β = 4.85, *P*-value = 4.44E-16) in FHS, adjusting for age, sex, smoking, alcohol, cell-type heterogeneity and family relationship, while DNA methylation at cg05639842 was not correlated with fasting glucose or insulin levels in the same model (Table 4). We found similar associations between cg06500161 methylation and fasting glucose (β = 0.853, *P*-value = 9.22E-7) and insulin levels (β = 4.05, *P*-value = 3.86E-21) in the WHI study (Table 4).

### Causal Effect of Statin Use on Type 2 Diabetes and Related Traits Through Epigenetics of *ABCG1* DNA Methylation

To determine the causal effect of statin use on type 2 diabetes risk/status and its related traits through DNA methylation at cg06500161, we conducted a two-step epigenetic Mendelian randomization (MR) approach in FHS and WHI consecutively. In the first step performed in FHS, SNPs were tested for associations with statin use and cg06500161 methylation, respectively, in (a) all participants and (b) non-diabetic participants. GSMR models then were implemented based on the summary results from GWAS, adjusting for sex, age, smoking, alcohol use, and cell-type heterogeneity. In this step, SNPs that were only associated with statin use were used to calculate a genetic score as the instrumental variable. For all participants, statin use was validated as a significant causal factor with cg06500161 methylation as the outcome (basic model: β = 0.0186, *P*-value = 2.56E-35; LDL-C and TG adjusted model: β = 0.0157, *P*-value = 1.05E-19; Table 5). We obtained similar results in the non-diabetic participants (basic model: β = 0.0179, *P*-value = 9.64E-31; LDL-C and TG adjusted model: β = 0.0142, *P*-value = 2.12E-19; Table 5).

**TABLE 5:** Two-step Mendelian randomization (MR) analysis in participants from the FHS and WHI studies.

		Exposure	Outcome	# SNP (Instrument variable)	β	SE	*P-*value	GWAS-threshold	MED effect via CpG methylation^‡^
***All participants***									
Step 1	FHS (Basic model)	Statin	cg06500161	200	0.0186	1.50E-3	2.56E-35	5E-5	
	FHS (LDL-C and TG adjusted)	Statin	cg06500161	162	0.0157	1.72E-3	1.05E-19	5E-5	
Step 2	WHI	cg06500161	T2D^†^	59	1.13	0.292	1.16E-4	5E-5	0.0177
***Non-diabetic participants***									
Step 1	FHS (Basic model)	Statin	cg06500161	177	0.0179	1.55E-3	9.64E-31	5E-5	
	FHS (LDL-C and TG adjusted)	Statin	cg06500161	171	0.0142	1.58E-3	2.12E-19	5E-5	
Step 2	WHI	cg06500161	Glucose	79	0.246	0.0693	3.87E-4	5E-5	3.49E-3
	WHI	cg06500161	Insulin	79	1.47	0.237	4.72E-10	5E-5	0.0209

*^†^T2D: Type 2 diabetes. ^‡^The causal effect of statin use on type 2 diabetes and related phenotypes through cg06500161 methylation was calculated by multiplying the genetic score estimates of step 2 MR by the genetic score estimate of step 1 MR in the LDL-C and TG adjusted model.*

In the second step of the MR analysis, conducted in WHI, we fitted a similar basic model for associations between cg06500161 methylation and type 2 diabetes in all participants, as well as cg06500161 and fasting glucose, cg06500161 and fasting insulin in the non-diabetic participants. Similar GSMR models were also implemented (diabetes: β = 1.13, *P*-value = 1.16E-4; glucose: β = 0.246, *P*-value = 3.87E-04; insulin: β = 1.47, *P*-value = 4.72E-10; Table 5). In this GSMR model, we used 59, 79 and 79 SNPs (Supplementary Table S1) as the instrumental variables in diabetes, glucose and insulin models, respectively. CpG cg06500161 methylation was validated as the causal factor with type 2 diabetes, glucose and insulin as the outcomes. Combining results from the first and second steps of MR, the causal effect of statins on type 2 diabetes, glucose, and insulin through cg06500161 methylation was calculated as 0.0177, 3.49E-3 and 0.0209, respectively (Table 5).

### Links Between Statin Use and DNA Methylation, and Gene Expression

To examine potential mechanisms through which statins mediated DNA methylation at cg06500161 that led to increased risk of diabetes, we separately correlated gene expression levels with statin use, cg06500161 methylation and fasting glucose and insulin levels. This was done with available gene expression data from all non-diabetic FHS participants. As shown in Supplementary Table S2, seventy-seven transcripts were correlated with both statin use and cg06500161 methylation. Fifty-six transcripts were found to be correlated with both fasting glucose and DNA methylation of cg06500161, and 80 transcripts were associated with both fasting insulin and cg06500161 methylation (Supplementary Table S2). Gene set enrichment analysis conducted with both KEGG and REACTOME gene-pathway assignments at an FDR of 10% suggested that the genes doubly associated with cg06500161 methylation and insulin are primarily involved in cholesterol and lipoprotein metabolic pathways (Table 6).

**TABLE 6:** Overlaps for doubly-associated gene expression in non-diabetic participants from the FHS study (genes in overlap ≥ 5, LDL-C and TG adjusted model).

Gene set name	Genes in overlap	*P-*value	FDR *q*-value
***Statin and CpG^†^***			
Reactome: NR1H2 and NR1H3-mediated signaling	5	2.70E-8	4.14E-5
Reactome: plasma lipoprotein assembly, remodeling, and clearance	5	2.22E-7	1.70E-4
Reactome: signaling by nuclear receptors	7	1.34E-6	6.86E-4
***CpG and Glucose***			
Reactome: metabolism of lipids and lipoproteins	5	5.49E-4	0.0787
***CpG and Insulin***			
Reactome: metabolism of lipids and lipoproteins	7	1.48E-5	6.38E-3
Reactome: transmembrane transport of small molecules	6	6.53E-5	0.0112

*^†^CpG: DNA methylation at cg06500161.*

**FIGURE 1:**
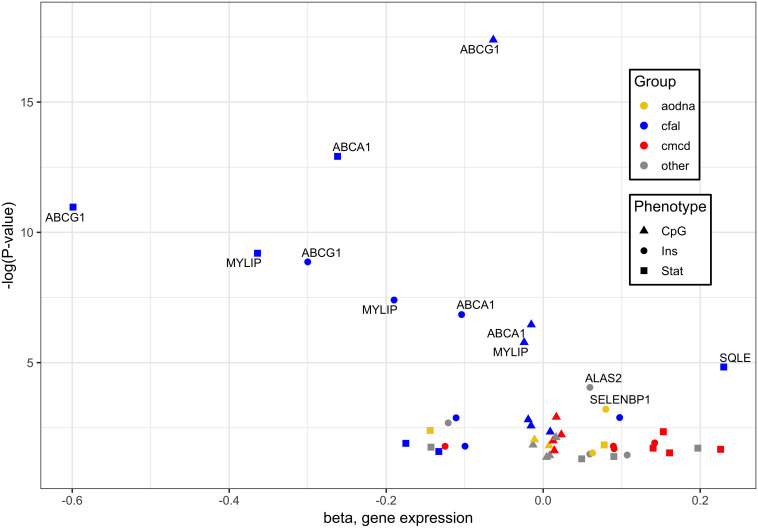
Genes whose expression associated significantly with cg06500161 methylation, glycemic traits and statin use. Of 16 genes whose expression in whole blood associated significantly with each of three different outcomes, those with the greatest responses are involved in cholesterol, fatty acid and lipid transport and homeostasis (blue). Associations were derived from methylation at *ABCG1* CpG cg06500161, glycemic factors, and statin use, designated by triangles, circles and squares, respectively. Other notable functional groups include cell migration and cytoskeletal dysnamics (red) and antioxidant defense and DNA damage response (orange). Plotted data taken from Supplementary Table S3.

When combining the transcripts whose expression in whole blood associated significantly with statin use, cg06500161 methylation and insulin level, 16 genes were found correlated with each of three different outcomes, with the greatest responses involved in cholesterol, fatty acid and lipid transport and homeostasis (Figure 1). Specifically, it should be noted that *ABCG1*, *ABCA1* and *ACSL3* were negatively, while *KAT2B* positively, associated with all signatures of statin use, CpG methylation and diabetes risk/status (Supplementary Tables S3, S4).

## Discussion

Our findings imply that statin use mediates DNA methylation at cg06500161 of *ABCG1* and this contributes to increased risk of type 2 diabetes. Firstly, we have shown that statin use was associated with DNA methylation modifications of *ABCG1* and such methylation patterns are correlated with the risk of type 2 diabetes. Secondly, Mendelian randomization demonstrates that statin use has moderate causal effects on type 2 diabetes and diabetes-related traits through differential methylation at cg06500161. Moreover, our results suggest that statin use and the methylation modifications in *ABCG1* are correlated with the expression of genes involved in both lipid metabolism and glycemic pathways.

This study reveals a potential epigenetic mechanism mediating the effect of statin use on glycemic signatures. The associations between epigenetic variants at *ABCG1* and diabetic traits have been observed previously (Hidalgo et al., 2014; Chambers et al., 2015; Dayeh et al., 2016), but little is known about the association between lipid-lowering medications and DNA methylation of *ABCG1*. Because DNA methylation at cg06500161 of *ABCG1* in blood has been associated positively with triglyceride levels and negatively with HDL-C levels (Dekkers et al., 2016; Braun et al., 2017), it is plausible that blood lipids have a causal effect on the epigenetic status at *ABCG1*. For this reason, in this study, we controlled for blood LDL cholesterol and triglyceride levels in our analyses. Importantly, our data from the LDL-C and TG adjusted model remained significant, indicating that statin use is an independent factor of higher cg06500161 methylation and consequently increased diabetic risk. Hence, the results from MR analysis have demonstrated that statin use is highly likely the causal factor of increased cg06500161 methylation and diabetic risk.

*ABCG1* encodes a protein member in the ATP-binding cassette (ABC) transporters superfamily, which transports specific intracellular sterols away from the endoplasmic reticulum (Tarling and Edwards, 2011). *ABCG1* has shown an allelic imbalance of expression in human pancreatic islet cells suggesting both genetic and epigenetic roles in altering the allelic expression of *ABCG1* (Serre et al., 2008). Indeed, genetic variants contribute to differences in *ABCG1* expression (Matsson et al., 2012). Does genetic variation impart stronger regulation of *ABCG1* expression than epigenetic modification? Previous GWAS have reported negative results in the associations between *ABCG1* and risk for type 2 diabetes (Vattikuti and Towler, 2004; Saxena et al., 2010). Coding SNPs in *ABCG1* were found not to be associated with risks for type 2 diabetes in the Copenhagen General Population Study (Schou et al., 2012). Frisdal et al. further demonstrated that *ABCG1* SNPs (rs1893590 and rs1378577) in severely obese individuals were not associated with the presence of diabetes or HOMA-IR (Frisdal et al., 2015). In the present study, we did not find any significant association between *ABCG1* SNPs with T2D, fasting glucose, or insulin levels in either FHS or WHI cohorts. The mechanisms of genetic variants on *ABCG1* and type 2 diabetes remain unclear and controversial, suggesting that epigenetic processes might be involved in the regulation of *ABCG1* and downstream gene expression, and ultimately diabetic signatures. Our results and those of others (Dayeh et al., 2016) show that increased DNA methylation at *ABCG1* was positively associated with diabetic signatures providing substantial evidence in support of these correlations between *ABCG1* and type 2 diabetes from an epigenetic perspective.

**FIGURE 2:**
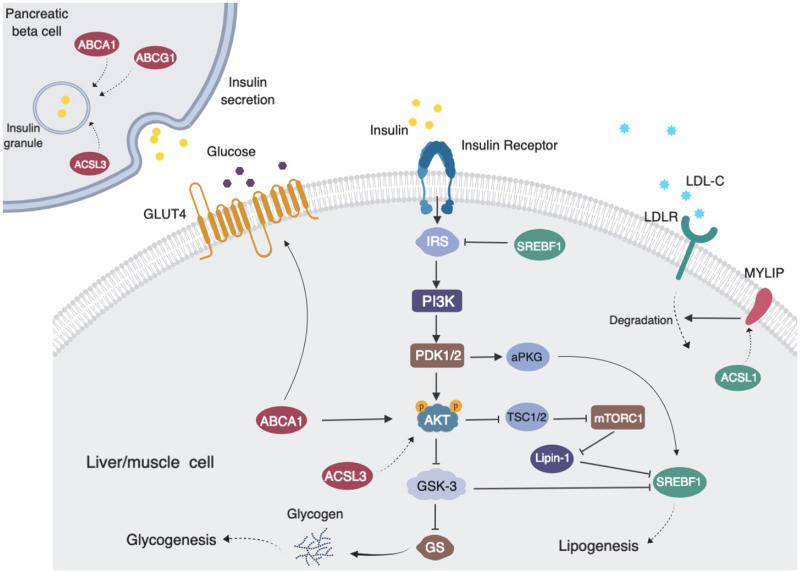
Proposed pathways of statin use associating with cg06500161 methylation, fasting blood glucose and fasting blood insulin levels (LDL-C and TG adjusted model). *ABCG1*, *ABCA1, ACSL3* and *MYLIP* (dark red) expression levels were negatively associated with medication use, CpG methylation and diabetic signatures. *SREBF1* and *ACSL3* (dark green) expression levels were inversely associated with medication use and CpG methylation. Statin use induced *ABCG1* methylation that leads to decreased expression of *ABCG1*, then altered insulin sensitivity and insulin secretion and increased risk of diabetes.

ABCG1 is a central regulator of cellular lipid homeostasis (Schmitz et al., 2001). Recently, several reports also suggest that ABCG1 may be involved in glucose metabolism. ABCG1 has been reported to mediate cholesterol transport and thus influence insulin secretion in pancreatic β-cells (Sturek et al., 2010; Kruit et al., 2012). Decreased *ABCG1* expression has been found in skeletal muscle from subjects with type 2 diabetes (Dayeh et al., 2016). In order to investigate possible methylation-driven gene expression changes and uncover the potential mechanism of diabetes risk mediated by statins, we examined the correlation of the transcriptome with statins, cg06500161 methylation status, and glucose and insulin levels in all non-diabetic participants in FHS. The expressions of *ABCG1, ABCA1, ACSL3* were negatively, while *KAT2B* positively, associated with the medication, cg06500161 methylation, and glucose and insulin levels. KAT2B (lysine acetyltransferase 2B), functions as a histone acetyltransferase to promote transcriptional activation (Ogryzko et al., 1996), has been reported to stimulate hepatic gluconeogenesis and increase circulating blood glucose concentrations, whereas inhibition of KAT2B improves glucose balance in insulin-resistant mice (Ravnskjaer et al., 2013). ABCA1 (ATP binding cassette subfamily A member 1), which normally moves cholesterol and phospholipids across cell membranes, could increase insulin secretion in pancreatic β-cells (Kruit et al., 2012). Additionally, silencing *ACSL3* and *ACSL4* (Long-chain acyl-CoA synthetases), which are responsible for activation of long-chain fatty acids, have been found to inhibit glucose-stimulated insulin release in pancreatic β-cells (Ansari et al., 2017). Meanwhile, previous findings, as illustrated in Figure 2, also have shown that reduced *ABCA1* and *ACSL3* expression impairs the phosphorylation of AKT, resulting in insulin resistance (de Haan et al., 2014; Li et al., 2018). Thus, decreased expression of *ABCG1*, *ABCA1*, and *ACSL3* could impair insulin secretion and lead to insulin resistance. All this supports the positive correlations observed here between statin use and insulin levels as well as glucose levels, which result in an increased risk of type 2 diabetes. It is also worth noting that other mechanisms such as epigenetic changes on non-coding RNAs and histone modifications might also affect the association between the statin use and T2D risk (Muka et al., 2016; Sun and Wong, 2016), and further studies are warranted to elucidate these mechanisms.

Some limitations should be acknowledged when interpreting the results of this study. First, there were differences in gender distribution between the two cohorts, in particular, WHI includes females only, which might contribute to slight inconsistencies in associations between statin use and methylation at *ABCG1*. Second, some glucose and insulin metabolism take place in the liver, pancreatic islet, adipose tissue, and skeletal muscle, but the available data permitted investigation of the mechanisms only in blood. This limitation in the sample type should not undermine the link between statin use and diabetes risk, as DNA methylation status in blood often reflects the epigenetic status in other tissues (Bysani et al., 2016; Crujeiras et al., 2017). Nevertheless, our findings suggest that further investigation is warranted in order to further characterize the molecular mechanism of increased diabetes risk with specific statin use.

In summary, we report DNA methylation site cg06500161 at *ABCG1* as a mediator of the association between statins and type 2 diabetes. These findings provide insights into the epigenetic mechanisms of statins influencing glycemic traits and further, type 2 diabetes pathogenesis. We show evidence for cg06500161 as a potential predictor or therapeutic target for prevention of type 2 diabetes. Implementation of functional studies of cg06500161 will uncover the precise molecular mechanisms. The eventual goal is to translate this information into clinical use, specifically, to develop useful prediction and prevention strategies for type 2 diabetes.

## Data Availability Statement

Publicly available datasets were analyzed in this study. These data can be found here: dbGaP: phs000200.v11.p3, phs000746.v2.p3, phs000342.v18.p11, phs000007.v29.p10, and phe00002.v6.

## Ethics Statement

The studies involving human participants were reviewed and approved by the Institutional Review Board (IRB) at Tufts University. The patients/participants provided their written informed consent to participate in this study.

## Author Contributions

C-QL and YL conceptualized and designed the study. YL, YS, TG, and LP contributed to data analysis. KW contributed to the processing and quality control of imputed genotypes of acquired through dbGaP. LP, JO, YS, TG, and CS critically evaluated the manuscript. JO is responsible for the funding and supervision of the study. YL and C-QL wrote and edited the manuscript. All authors read and approved the final manuscript.

## Conflict of Interest

The authors declare that the research was conducted in the absence of any commercial or financial relationships that could be construed as a potential conflict of interest.

## Footnotes

1 https://www.cog-genomics.org/plink/2.0/
