# Supplementary material for: Statin Use Associates With Risk of Type 2 Diabetes via Epigenetic Patterns at ABCG1
Source: Front Genet. 2020 Jun 16;11:622. doi: 10.3389/fgene.2020.00622 (PMC7308584; doi:10.3389/fgene.2020.00622)
Supplement: Supplementary file 1 [file Data_Sheet_1.docx]

**Table S1.** SNPs used to construct genetic scores as the instrumental variable (*P-*value < 5E-5 and LD r^2^ < 0.05) in the two-step Mendelian randomization (MR) analysis in participants from the WHI study.

| SNP | Gene | A1 | A2 | Frequency | β_exposure | SE_exposure | β_outcome | SE_outcome |
| --- | --- | --- | --- | --- | --- | --- | --- | --- |
| ***CpG*-T2D*^†^ *model* (n = 59)** | | | | | | | | |
| rs10024219 | EMCN | A | G | 0.84540 | -0.00753 | 0.00180 | -0.01531 | 0.01472 |
| rs10061061 | LINC01331 | T | C | 0.38237 | 0.00582 | 0.00134 | 0.00348 | 0.01100 |
| rs10076899 | - | G | C | 0.77947 | -0.00659 | 0.00156 | 0.00004 | 0.01280 |
| rs10083144 | - | C | T | 0.82497 | -0.00708 | 0.00173 | -0.02951 | 0.01415 |
| rs10229483 | TAX1BP1 | A | T | 0.58557 | -0.00548 | 0.00131 | 0.00077 | 0.01072 |
| rs1076733 | GLP1R | A | G | 0.46070 | 0.00559 | 0.00128 | -0.00202 | 0.01049 |
| rs10836266 | EHF | C | G | 0.54085 | 0.00548 | 0.00131 | 0.02784 | 0.01072 |
| rs10846803 | BRI3BP | C | T | 0.73578 | -0.00669 | 0.00152 | -0.02783 | 0.01248 |
| rs10848098 | RIMBP2 | A | G | 0.51267 | -0.00543 | 0.00133 | -0.01106 | 0.01091 |
| rs112215807 | - | G | T | 0.93175 | 0.01020 | 0.00251 | 0.02101 | 0.02067 |
| rs112730388 | - | A | G | 0.85677 | 0.00894 | 0.00184 | 0.01273 | 0.01502 |
| rs115099294 | MICAL1 | G | A | 0.94442 | 0.01117 | 0.00275 | -0.00727 | 0.02256 |
| rs12438636 | AC087490.1 | C | T | 0.89581 | -0.00888 | 0.00212 | -0.01984 | 0.01745 |
| rs12649504 | AC098829.1 | T | C | 0.37565 | -0.00621 | 0.00136 | -0.01099 | 0.01109 |
| rs12671202 | - | A | G | 0.15590 | -0.00830 | 0.00180 | -0.03812 | 0.01479 |
| rs12692401 | HPCAL1 | C | G | 0.25982 | 0.00590 | 0.00145 | 0.00187 | 0.01196 |
| rs13156777 | - | T | A | 0.82885 | -0.00848 | 0.00175 | -0.00150 | 0.01422 |
| rs142587282 | CSMD1 | C | T | 0.89478 | 0.00856 | 0.00209 | -0.00541 | 0.01718 |
| rs1460796 | LINC02694 | G | C | 0.81722 | -0.00756 | 0.00169 | -0.03343 | 0.01390 |
| rs147698241 | MYO1D | C | T | 0.94933 | -0.01257 | 0.00303 | -0.01628 | 0.02463 |
| rs148538153 | FARP2 | A | T | 0.91908 | -0.01025 | 0.00239 | 0.03225 | 0.01967 |
| rs17134799 | - | G | C | 0.92011 | 0.00974 | 0.00233 | -0.02954 | 0.01919 |
| rs183271700 | EMILIN2 | A | G | 0.56334 | -0.00548 | 0.00135 | -0.01873 | 0.01105 |
| rs1865301 | - | A | T | 0.52585 | -0.00541 | 0.00130 | -0.00346 | 0.01066 |
| rs2051719 | SV2B | G | C | 0.85858 | -0.00758 | 0.00186 | -0.01444 | 0.01530 |
| rs225443 | ABCG1 | A | G | 0.38806 | -0.00642 | 0.00132 | 0.01853 | 0.01089 |
| rs233295 | TSPEAR | T | C | 0.87849 | -0.00872 | 0.00212 | 0.01656 | 0.01724 |
| rs2424475 | - | C | T | 0.82006 | -0.00686 | 0.00167 | -0.00794 | 0.01374 |
| rs255824 | SPDL1 | C | T | 0.17658 | 0.00744 | 0.00171 | 0.02563 | 0.01398 |
| rs2675416 | P3H2-AS1 | T | A | 0.73242 | -0.00727 | 0.00146 | -0.01014 | 0.01202 |
| rs2705052 | SLC7A2 | A | G | 0.35755 | 0.00608 | 0.00137 | 0.00982 | 0.01124 |
| rs28390137 | MMD | C | T | 0.93873 | 0.01116 | 0.00272 | 0.02894 | 0.02234 |
| rs2844420 | ITGA9 | G | A | 0.75621 | -0.00662 | 0.00152 | -0.00789 | 0.01252 |
| rs28485889 | - | C | G | 0.88883 | -0.00840 | 0.00207 | 0.00280 | 0.01695 |
| rs2996231 | - | G | T | 0.83635 | -0.00830 | 0.00181 | -0.03425 | 0.01477 |
| rs34527859 | ALDH4A1 | C | G | 0.91986 | 0.00994 | 0.00238 | 0.02752 | 0.01964 |
| rs4363999 | MYO3B | T | G | 0.57601 | 0.00529 | 0.00128 | 0.01714 | 0.01056 |
| rs458347 | - | T | C | 0.39581 | 0.00565 | 0.00137 | -0.00125 | 0.01126 |
| rs484681 | AC034114.2 | A | G | 0.83583 | 0.00748 | 0.00173 | -0.00078 | 0.01424 |
| rs4937304 | LINC02725 | C | G | 0.80093 | 0.00682 | 0.00160 | 0.00768 | 0.01316 |
| rs4947185 | RPSAP45 | C | T | 0.38728 | 0.00543 | 0.00133 | 0.01821 | 0.01084 |
| rs4977592 | AL391117.1 | G | A | 0.58583 | -0.00608 | 0.00134 | 0.00776 | 0.01103 |
| rs4980047 | ZMIZ1 | A | G | 0.24354 | 0.00693 | 0.00156 | 0.02784 | 0.01273 |
| rs56332954 | MIR548XHG | A | G | 0.73345 | 0.00601 | 0.00146 | -0.00077 | 0.01200 |
| rs62295401 | - | G | A | 0.94364 | -0.01179 | 0.00280 | -0.06326 | 0.02312 |
| rs62505318 | PXDNL | C | T | 0.90072 | 0.00972 | 0.00224 | 0.00378 | 0.01832 |
| rs6482594 | ANKRD26 | C | T | 0.80636 | 0.00717 | 0.00167 | 0.01799 | 0.01367 |
| rs7036196 | MEGF9 | A | G | 0.37384 | 0.00582 | 0.00137 | -0.00064 | 0.01115 |
| rs73533289 | - | G | A | 0.94674 | -0.01194 | 0.00288 | -0.00632 | 0.02361 |
| rs752208 | DNMT3A | A | G | 0.23268 | 0.00730 | 0.00161 | -0.00590 | 0.01319 |
| rs77215255 | LINC01592 | T | C | 0.93847 | 0.01374 | 0.00268 | 0.01468 | 0.02209 |
| rs9420343 | TACC2 | G | A | 0.52740 | 0.00531 | 0.00130 | 0.01474 | 0.01068 |
| rs9983344 | ABCG1 | C | T | 0.90874 | -0.02522 | 0.00229 | 0.00724 | 0.01865 |
| 13:21154866:T_TC |  | 7 | 5 | 0.92865 | -0.01042 | 0.00252 | 0.00755 | 0.02071 |
| 15:50527201:AG_A |  | 6 | 7 | 0.11918 | -0.01037 | 0.00208 | -0.01957 | 0.01704 |
| 2:121865336:TTA_T |  | 7 | 6 | 0.63263 | -0.00685 | 0.00152 | -0.03036 | 0.01219 |
| 21:43570419:TCA_T |  | 7 | 6 | 0.79447 | -0.00671 | 0.00164 | 0.01622 | 0.01342 |
| 4:60829708:T_TTT |  | 7 | 5 | 0.93873 | -0.01169 | 0.00275 | -0.02829 | 0.02241 |
| 4:62377855:GT_G |  | 6 | 7 | 0.70915 | 0.00598 | 0.00141 | -0.01102 | 0.01160 |
| ***CpG-Glucose model* (n = 79)** | | | | | | | | |
| rs10024219 | EMCN | A | G | 0.84914 | -0.00862 | 0.00191 | -0.00545 | 0.00420 |
| rs10444501 | KSR2 | T | A | 0.84114 | -0.00750 | 0.00181 | 0.00221 | 0.00410 |
| rs10514220 | SSBP2 | A | G | 0.89478 | 0.00949 | 0.00222 | 0.00537 | 0.00500 |
| rs1076733 | GLP1R | A | G | 0.46147 | 0.00549 | 0.00135 | 0.00271 | 0.00307 |
| rs10931042 | FRZB | G | T | 0.75845 | 0.00655 | 0.00158 | 0.00311 | 0.00355 |
| rs1094723 | - | C | T | 0.32424 | -0.00614 | 0.00151 | -0.00426 | 0.00328 |
| rs11080536 | IMPA2 | A | G | 0.89656 | -0.00921 | 0.00226 | -0.00338 | 0.00495 |
| rs111602846 | CNTN4 | C | T | 0.88204 | -0.00853 | 0.00210 | 0.00006 | 0.00460 |
| rs112642128 | PGC | C | T | 0.11855 | 0.00897 | 0.00208 | 0.00348 | 0.00467 |
| rs112730388 | - | A | G | 0.85270 | 0.00901 | 0.00191 | 0.00400 | 0.00427 |
| rs11609954 | BRI3BP | A | G | 0.27890 | 0.00684 | 0.00159 | -0.00011 | 0.00359 |
| rs11641583 | LINC02140 | A | C | 0.57795 | -0.00657 | 0.00156 | 0.00039 | 0.00331 |
| rs116728255 | AC007106.1 | C | G | 0.94369 | 0.01266 | 0.00303 | 0.00269 | 0.00619 |
| rs1202199 | MBOAT1 | T | C | 0.13545 | -0.00833 | 0.00203 | -0.00721 | 0.00453 |
| rs12083070 | - | C | T | 0.89953 | 0.00916 | 0.00223 | 0.00634 | 0.00485 |
| rs1235407 | ACYP2 | A | G | 0.46117 | 0.00605 | 0.00139 | 0.00556 | 0.00303 |
| rs12649504 | AC098829.1 | T | C | 0.38234 | -0.00632 | 0.00143 | 0.00546 | 0.00311 |
| rs12692401 | HPCAL1 | C | G | 0.25874 | 0.00683 | 0.00154 | -0.00017 | 0.00353 |
| rs13155995 | - | G | A | 0.37789 | 0.00595 | 0.00144 | -0.00681 | 0.00322 |
| rs13156777 | - | T | A | 0.82691 | -0.00896 | 0.00183 | 0.00046 | 0.00401 |
| rs1329571 | PAX5 | A | T | 0.54268 | 0.00601 | 0.00138 | 0.00039 | 0.00301 |
| rs139176105 | OSMR | C | T | 0.94517 | 0.01327 | 0.00307 | 0.00282 | 0.00672 |
| rs144785530 | - | C | A | 0.68198 | -0.00640 | 0.00147 | -0.00242 | 0.00326 |
| rs1460796 | LINC02694 | G | C | 0.82484 | -0.00813 | 0.00183 | -0.00037 | 0.00405 |
| rs148538153 | FARP2 | A | T | 0.91731 | -0.01078 | 0.00251 | -0.00732 | 0.00566 |
| rs17134799 | - | G | C | 0.92205 | 0.01022 | 0.00251 | -0.00411 | 0.00570 |
| rs17149572 | CELF2 | C | T | 0.90842 | -0.01034 | 0.00243 | 0.00479 | 0.00523 |
| rs2192510 | LMBR1 | C | T | 0.91909 | -0.01043 | 0.00254 | -0.00151 | 0.00580 |
| rs225443 | ABCG1 | A | G | 0.38056 | -0.00638 | 0.00141 | 0.00043 | 0.00314 |
| rs233295 | TSPEAR | T | C | 0.88026 | -0.00947 | 0.00224 | -0.00395 | 0.00457 |
| rs2348113 | AOX3P | C | G | 0.91553 | -0.01066 | 0.00248 | -0.00283 | 0.00542 |
| rs2499900 | AKAP12 | A | G | 0.14286 | 0.00777 | 0.00191 | 0.00299 | 0.00433 |
| rs2574 | DGKG | C | G | 0.51037 | 0.00567 | 0.00137 | 0.00456 | 0.00313 |
| rs2798750 | - | G | A | 0.79668 | -0.00717 | 0.00172 | -0.00528 | 0.00379 |
| rs2922666 | - | G | T | 0.81506 | 0.00751 | 0.00178 | 0.00532 | 0.00400 |
| rs2929946 | CCN4 | G | A | 0.59010 | -0.00640 | 0.00141 | -0.00286 | 0.00319 |
| rs3111851 | P3H2-AS1 | G | C | 0.74481 | -0.00784 | 0.00156 | -0.00015 | 0.00353 |
| rs34527859 | ALDH4A1 | C | G | 0.91701 | 0.01130 | 0.00248 | 0.00949 | 0.00568 |
| rs35981794 | - | T | C | 0.83966 | 0.00804 | 0.00193 | 0.00895 | 0.00426 |
| rs4450197 | CADM1 | C | T | 0.08625 | 0.01026 | 0.00250 | 0.00203 | 0.00530 |
| rs4462101 | - | G | A | 0.89004 | -0.00910 | 0.00221 | 0.00134 | 0.00493 |
| rs4771763 | AL359649.1 | A | G | 0.64641 | -0.00576 | 0.00141 | 0.00052 | 0.00320 |
| rs4791313 | NDEL1 | C | T | 0.61085 | 0.00578 | 0.00137 | 0.00254 | 0.00313 |
| rs484681 | AC034114.2 | A | G | 0.83462 | 0.00777 | 0.00182 | 0.00540 | 0.00414 |
| rs4947185 | RPSAP45 | C | T | 0.38530 | 0.00617 | 0.00141 | 0.00354 | 0.00311 |
| rs4977592 | AL391117.1 | G | A | 0.58121 | -0.00727 | 0.00141 | -0.00208 | 0.00321 |
| rs55986143 | AL589740.1 | T | G | 0.94191 | -0.01206 | 0.00296 | 0.00017 | 0.00668 |
| rs56984394 | - | G | T | 0.52875 | 0.00590 | 0.00138 | -0.00317 | 0.00310 |
| rs57467996 | - | T | G | 0.91583 | -0.01025 | 0.00244 | -0.00425 | 0.00549 |
| rs58533449 | DLG2 | A | G | 0.90723 | 0.00976 | 0.00237 | 0.01142 | 0.00508 |
| rs60527674 | JAZF1 | A | G | 0.88026 | -0.00903 | 0.00219 | -0.00547 | 0.00467 |
| rs61424529 | COL14A1 | C | G | 0.93983 | 0.01393 | 0.00315 | 0.00188 | 0.00608 |
| rs61901845 | - | C | T | 0.90427 | 0.00968 | 0.00237 | 0.00596 | 0.00528 |
| rs61928479 | ABCC9 | T | A | 0.90012 | -0.00935 | 0.00224 | -0.00137 | 0.00513 |
| rs61975704 | - | C | A | 0.92442 | -0.01093 | 0.00261 | -0.00655 | 0.00605 |
| rs62505318 | PXDNL | C | T | 0.89804 | 0.01017 | 0.00233 | -0.00666 | 0.00520 |
| rs6480322 | - | T | A | 0.85418 | 0.00819 | 0.00201 | -0.00021 | 0.00407 |
| rs6770624 | THRB-AS1 | C | T | 0.18287 | 0.00737 | 0.00179 | -0.00705 | 0.00393 |
| rs7018535 | - | A | C | 0.76408 | -0.00689 | 0.00163 | -0.00110 | 0.00360 |
| rs7036196 | MEGF9 | A | G | 0.37107 | 0.00620 | 0.00144 | -0.00204 | 0.00312 |
| rs73218555 | - | A | T | 0.78720 | 0.00692 | 0.00168 | 0.00473 | 0.00365 |
| rs7514206 | NBPF13P | G | A | 0.88530 | -0.00891 | 0.00218 | -0.00200 | 0.00470 |
| rs76108595 | TMEM132C | G | A | 0.93450 | -0.01240 | 0.00296 | -0.00433 | 0.00599 |
| rs77215255 | LINC01592 | T | C | 0.93657 | 0.01253 | 0.00280 | 0.00528 | 0.00631 |
| rs798449 | LINC00298 | A | G | 0.58062 | 0.00561 | 0.00138 | 0.00116 | 0.00314 |
| rs881558 | LINC01700 | G | A | 0.90456 | -0.00977 | 0.00236 | -0.00311 | 0.00518 |
| rs9420343 | TACC2 | G | A | 0.52460 | 0.00648 | 0.00138 | 0.00647 | 0.00313 |
| rs9983344 | ABCG1 | C | T | 0.91049 | -0.02398 | 0.00246 | -0.00017 | 0.00525 |
| 10:5029283 | - | G | A | 0.88204 | 0.00890 | 0.00218 | 0.00884 | 0.00503 |
| 15:50527201:AG_A | - | 6 | 7 | 0.12389 | -0.01101 | 0.00217 | 0.00328 | 0.00488 |
| 16:5538550:G_GTG | - | 7 | 5 | 0.47392 | 0.00637 | 0.00149 | -0.00453 | 0.00313 |
| 17:56265684:GC_G | - | 6 | 7 | 0.07410 | 0.01128 | 0.00263 | 0.00000 | 0.00565 |
| 19:34071615:C_CT | - | 5 | 7 | 0.53379 | -0.00583 | 0.00142 | -0.00279 | 0.00298 |
| 4:162848512:A_AT | - | 7 | 5 | 0.89478 | 0.00964 | 0.00232 | 0.00469 | 0.00478 |
| 4:41198045:C_CG | - | 5 | 7 | 0.15471 | 0.00806 | 0.00194 | 0.00543 | 0.00428 |
| 4:62377855:GT_G | - | 6 | 7 | 0.71043 | 0.00612 | 0.00149 | -0.00272 | 0.00339 |
| 7:49626201:A_AT | - | 5 | 7 | 0.88945 | -0.00959 | 0.00218 | -0.00353 | 0.00491 |
| 7:69747056:A_ATG | - | 7 | 5 | 0.90931 | -0.01185 | 0.00245 | 0.00125 | 0.00537 |
| 9:107777631:T_TC | - | 7 | 5 | 0.93302 | 0.01128 | 0.00277 | 0.00358 | 0.00626 |
| ***CpG-Insulin model* (n = 79)** | | | | | | | | |
| rs10024219 | EMCN | A | G | 0.84914 | -0.00862 | 0.00191 | 0.00141 | 0.01416 |
| rs10444501 | KSR2 | T | A | 0.84114 | -0.00750 | 0.00181 | -0.01256 | 0.01347 |
| rs10514220 | SSBP2 | A | G | 0.89478 | 0.00949 | 0.00222 | -0.00929 | 0.01653 |
| rs1076733 | GLP1R | A | G | 0.46147 | 0.00549 | 0.00135 | 0.01245 | 0.01007 |
| rs10931042 | FRZB | G | T | 0.75845 | 0.00655 | 0.00158 | -0.00719 | 0.01176 |
| rs1094723 | - | C | T | 0.32424 | -0.00614 | 0.00151 | -0.01583 | 0.01114 |
| rs11080536 | IMPA2 | A | G | 0.89656 | -0.00921 | 0.00226 | -0.02233 | 0.01687 |
| rs111602846 | CNTN4 | C | T | 0.88204 | -0.00853 | 0.00210 | 0.01824 | 0.01550 |
| rs112642128 | PGC | C | T | 0.11855 | 0.00897 | 0.00208 | 0.00356 | 0.01561 |
| rs112730388 | - | A | G | 0.85270 | 0.00901 | 0.00191 | 0.02728 | 0.01417 |
| rs11609954 | BRI3BP | A | G | 0.27890 | 0.00684 | 0.00159 | -0.00568 | 0.01183 |
| rs11641583 | LINC02140 | A | C | 0.57795 | -0.00657 | 0.00156 | -0.01159 | 0.01157 |
| rs116728255 | AC007106.1 | C | G | 0.94369 | 0.01266 | 0.00303 | 0.03999 | 0.02265 |
| rs1202199 | MBOAT1 | T | C | 0.13545 | -0.00833 | 0.00203 | 0.00648 | 0.01508 |
| rs12083070 | - | C | T | 0.89953 | 0.00916 | 0.00223 | 0.00823 | 0.01677 |
| rs1235407 | ACYP2 | A | G | 0.46117 | 0.00605 | 0.00139 | 0.01608 | 0.01031 |
| rs12649504 | AC098829.1 | T | C | 0.38234 | -0.00632 | 0.00143 | -0.00380 | 0.01061 |
| rs12692401 | HPCAL1 | C | G | 0.25874 | 0.00683 | 0.00154 | 0.02160 | 0.01139 |
| rs13155995 | - | G | A | 0.37789 | 0.00595 | 0.00144 | 0.00657 | 0.01074 |
| rs13156777 | - | T | A | 0.82691 | -0.00896 | 0.00183 | 0.01014 | 0.01360 |
| rs1329571 | PAX5 | A | T | 0.54268 | 0.00601 | 0.00138 | 0.01959 | 0.01028 |
| rs139176105 | OSMR | C | T | 0.94517 | 0.01327 | 0.00307 | 0.04302 | 0.02278 |
| rs144785530 | - | C | A | 0.68198 | -0.00640 | 0.00147 | 0.00005 | 0.01091 |
| rs1460796 | LINC02694 | G | C | 0.82484 | -0.00813 | 0.00183 | -0.01976 | 0.01362 |
| rs148538153 | FARP2 | A | T | 0.91731 | -0.01078 | 0.00251 | -0.03355 | 0.01878 |
| rs17134799 | - | G | C | 0.92205 | 0.01022 | 0.00251 | 0.02695 | 0.01851 |
| rs17149572 | CELF2 | C | T | 0.90842 | -0.01034 | 0.00243 | -0.01974 | 0.01807 |
| rs2192510 | LMBR1 | C | T | 0.91909 | -0.01043 | 0.00254 | -0.01737 | 0.01883 |
| rs225443 | ABCG1 | A | G | 0.38056 | -0.00638 | 0.00141 | 0.00971 | 0.01050 |
| rs233295 | TSPEAR | T | C | 0.88026 | -0.00947 | 0.00224 | -0.00083 | 0.01655 |
| rs2348113 | AOX3P | C | G | 0.91553 | -0.01066 | 0.00248 | -0.02123 | 0.01848 |
| rs2499900 | AKAP12 | A | G | 0.14286 | 0.00777 | 0.00191 | 0.06197 | 0.01420 |
| rs2574 | DGKG | C | G | 0.51037 | 0.00567 | 0.00137 | 0.00969 | 0.01020 |
| rs2798750 | - | G | A | 0.79668 | -0.00717 | 0.00172 | -0.00215 | 0.01280 |
| rs2922666 | - | G | T | 0.81506 | 0.00751 | 0.00178 | 0.02083 | 0.01320 |
| rs2929946 | CCN4 | G | A | 0.59010 | -0.00640 | 0.00141 | 0.00364 | 0.01042 |
| rs3111851 | P3H2-AS1 | G | C | 0.74481 | -0.00784 | 0.00156 | -0.02616 | 0.01155 |
| rs34527859 | ALDH4A1 | C | G | 0.91701 | 0.01130 | 0.00248 | 0.03211 | 0.01851 |
| rs35981794 | - | T | C | 0.83966 | 0.00804 | 0.00193 | 0.02062 | 0.01430 |
| rs4450197 | CADM1 | C | T | 0.08625 | 0.01026 | 0.00250 | 0.01205 | 0.01865 |
| rs4462101 | - | G | A | 0.89004 | -0.00910 | 0.00221 | -0.01661 | 0.01643 |
| rs4771763 | AL359649.1 | A | G | 0.64641 | -0.00576 | 0.00141 | -0.00573 | 0.01045 |
| rs4791313 | NDEL1 | C | T | 0.61085 | 0.00578 | 0.00137 | 0.00998 | 0.01020 |
| rs484681 | AC034114.2 | A | G | 0.83462 | 0.00777 | 0.00182 | 0.01799 | 0.01352 |
| rs4947185 | RPSAP45 | C | T | 0.38530 | 0.00617 | 0.00141 | 0.01882 | 0.01042 |
| rs4977592 | AL391117.1 | G | A | 0.58121 | -0.00727 | 0.00141 | -0.00718 | 0.01048 |
| rs55986143 | AL589740.1 | T | G | 0.94191 | -0.01206 | 0.00296 | -0.00748 | 0.02196 |
| rs56984394 | - | G | T | 0.52875 | 0.00590 | 0.00138 | 0.00757 | 0.01028 |
| rs57467996 | - | T | G | 0.91583 | -0.01025 | 0.00244 | -0.01201 | 0.01812 |
| rs58533449 | DLG2 | A | G | 0.90723 | 0.00976 | 0.00237 | 0.02955 | 0.01764 |
| rs60527674 | JAZF1 | A | G | 0.88026 | -0.00903 | 0.00219 | -0.03503 | 0.01636 |
| rs61424529 | COL14A1 | C | G | 0.93983 | 0.01393 | 0.00315 | 0.00644 | 0.02331 |
| rs61901845 | - | C | T | 0.90427 | 0.00968 | 0.00237 | 0.00219 | 0.01757 |
| rs61928479 | ABCC9 | T | A | 0.90012 | -0.00935 | 0.00224 | -0.02364 | 0.01675 |
| rs61975704 | - | C | A | 0.92442 | -0.01093 | 0.00261 | -0.02610 | 0.01935 |
| rs62505318 | PXDNL | C | T | 0.89804 | 0.01017 | 0.00233 | 0.00148 | 0.01732 |
| rs6480322 | - | T | A | 0.85418 | 0.00819 | 0.00201 | -0.00409 | 0.01494 |
| rs6770624 | THRB-AS1 | C | T | 0.18287 | 0.00737 | 0.00179 | 0.02028 | 0.01338 |
| rs7018535 | - | A | C | 0.76408 | -0.00689 | 0.00163 | -0.01672 | 0.01215 |
| rs7036196 | MEGF9 | A | G | 0.37107 | 0.00620 | 0.00144 | 0.01952 | 0.01072 |
| rs73218555 | - | A | T | 0.78720 | 0.00692 | 0.00168 | 0.03869 | 0.01248 |
| rs7514206 | NBPF13P | G | A | 0.88530 | -0.00891 | 0.00218 | -0.02087 | 0.01616 |
| rs76108595 | TMEM132C | G | A | 0.93450 | -0.01240 | 0.00296 | -0.01629 | 0.02189 |
| rs77215255 | LINC01592 | T | C | 0.93657 | 0.01253 | 0.00280 | 0.01922 | 0.02070 |
| rs798449 | LINC00298 | A | G | 0.58062 | 0.00561 | 0.00138 | 0.01650 | 0.01023 |
| rs881558 | LINC01700 | G | A | 0.90456 | -0.00977 | 0.00236 | -0.01007 | 0.01757 |
| rs9420343 | TACC2 | G | A | 0.52460 | 0.00648 | 0.00138 | 0.00978 | 0.01022 |
| rs9983344 | ABCG1 | C | T | 0.91049 | -0.02398 | 0.00246 | -0.00156 | 0.01830 |
| 10:5029283 | - | G | A | 0.88204 | 0.00890 | 0.00218 | 0.01028 | 0.01631 |
| 15:50527201:AG_A | - | 6 | 7 | 0.12389 | -0.01101 | 0.00217 | -0.01423 | 0.01615 |
| 16:5538550:G_GTG | - | 7 | 5 | 0.47392 | 0.00637 | 0.00149 | 0.01253 | 0.01105 |
| 17:56265684:GC_G | - | 6 | 7 | 0.07410 | 0.01128 | 0.00263 | -0.02249 | 0.01959 |
| 19:34071615:C_CT | - | 5 | 7 | 0.53379 | -0.00583 | 0.00142 | -0.01578 | 0.01058 |
| 4:162848512:A_AT | - | 7 | 5 | 0.89478 | 0.00964 | 0.00232 | 0.00277 | 0.01722 |
| 4:41198045:C_CG | - | 5 | 7 | 0.15471 | 0.00806 | 0.00194 | 0.02715 | 0.01442 |
| 4:62377855:GT_G | - | 6 | 7 | 0.71043 | 0.00612 | 0.00149 | 0.00751 | 0.01107 |
| 7:49626201:A_AT | - | 5 | 7 | 0.88945 | -0.00959 | 0.00218 | -0.03208 | 0.01617 |
| 7:69747056:A_ATG | - | 7 | 5 | 0.90931 | -0.01185 | 0.00245 | -0.03379 | 0.01807 |
| 9:107777631:T_TC | - | 7 | 5 | 0.93302 | 0.01128 | 0.00277 | -0.02588 | 0.02061 |

* CpG: cg06500161; † T2D: Type 2 diabetes.

**Table S2.** Gene expression associated with two traits of statin use (Stat), cg06500161 methylation (CpG) and fasting insulin (Ins)/fasting glucose (Glu) in non-diabetic participants from the FHS study (*P* < 0.05, lipids-adjusted model).

| Stat and CpG | CpG and Ins | CpG and Glu |
| --- | --- | --- |
| *LOC731508* | *RTCD1* | *RTCD1* |
| *PRRX1* | *STXBP3* | *NUF2* |
| *TRIM58* | *SLC22A15* | *PFKFB2* |
| *PAFAH2* | *PFKFB2* | *MARK1* |
| *SELENBP1* | *MARK1* | *FAM69A* |
| *SELP* | *FCRL1* | *GOLPH3L* |
| *ARPC5* | *FAM129A* | *ACBD3* |
| *MT1H* | *TAF5L* | *ZNF496* |
| *MERTK* | *UNQ9419* | *QPCT* |
| *MYO7B* | *IL18R1* | *UNQ9419* |
| *MYO1B* | *TTLL4* | *KCNH7* |
| *TTLL4* | *CYP27A1* | *FN1* |
| *LOC285181* | *DPP4* | *CACNA1D* |
| *ACSL3* | *FN1* | *C3orf37* |
| *IK* | *FANCD2* | *SENP2* |
| *KAT2B* | *SENP2* | *HEG1* |
| *CD96* | *HEG1* | *ZBBX* |
| *LEKR1* | *UBXN7* | *AGA* |
| *SETD2* | *SRD5A3* | *ACSL1* |
| *DNAJC8* | *FLJ45721* | *PPWD1* |
| *BST1* | *PRDM5* | *TMEM174* |
| *TAPT1* | *PDGFC* | *VCAN* |
| *ELOVL6* | *ACSL1* | *UBE2D2* |
| *C4orf31* | *TUBB4Q* | *HLA-DPB1* |
| *MEF2C* | *VCAN* | *CREB5* |
| *MYLIP* | *NBPF22P* | *TFEC* |
| *AKAP12* | *HTR4* | *KBTBD11* |
| *IGF2R* | *HAVCR2* | *TMEM71* |
| *RAET1E* | *HLA-DPB1* | *ADFP* |
| *EPB49* | *FLJ43093* | *C10orf54* |
| *PKHD1L1* | *AKAP12* | *GSTP1* |
| *SQLE* | *IGF2R* | *ANO1* |
| *ANK1* | *CREB5* | *OR52H1* |
| *PSIP1* | *ABCA13* | *CPT1A* |
| *WDR40A* | *FGL2* | *MMP8* |
| *ABCA1* | *CDK6* | *ZNF641* |
| *ST6GALNAC4* | *ZDHHC2* | *DDX23* |
| *ZER1* | *DEFA4* | *HVCN1* |
| *ADK* | *LRRC6* | *UBAC2* |
| *RSU1* | *UHRF2* | *TDP1* |
| *C10orf67* | *NFIB* | *SERPINA1* |
| *SVIL* | *ALDH1A1* | *BAIAP3* |
| *CUZD1* | *PLXDC2* | *FLYWCH1* |
| *MICAL2* | *GSTO2* | *ATXN2L* |
| *GLYATL1* | *SLC16A9* | *DLG4* |
| *C11orf84* | *CPT1A* | *LDLR* |
| *ACADS* | *MMP8* | *GNG7* |
| *A2M* | *MUCL1* | *B4GALT5* |
| *PZP* | *TMTC2* | *ACADVL* |
| *EPS8* | *PLXNC1* | *ABCA1* |
| *PTGES3* | *MLEC* | *ABCG1* |
| *PPTC7* | *FLJ22662* | *ACSL3* |
| *SLC15A4* | *TRPV4* | *IL17REL* |
| *TFDP1* | *MAPK6* | *MEF2C* |
| *C14orf37* | *CILP* | *SELENBP1* |
| *SPTB* | *ATXN2L* | *SPTB* |
| *CINP* | *haptoglobin* |  |
| *PML* | *AFG3L1* |  |
| *ITGAL* | *SAP30BP* |  |
| *MT4* | *TNRC6C* |  |
| *PLA2G15* | *SERPINB10* |  |
| *LOC100129677* | *LDLR* |  |
| *CYTSB* | *CEACAM8* |  |
| *SREBF1* | *TMEM160* |  |
| *ULK2* | *C20orf7* |  |
| *FAM117A* | *BPI* |  |
| *MKS1* | *ICOSLG* |  |
| *APOC4* | *PANX2* |  |
| *PHLDB3* | *ABCA1* |  |
| *LOC100130077* | *ABCG1* |  |
| *ABCG1* | *ACSL3* |  |
| *TRABD* | *BST2* |  |
| *IL17REL* | *EPS8* |  |
| *REPS2* | *GZMB* |  |
| *LANCL3* | *KAT2B* |  |
| *UTX* | *MEF2C* |  |
| *ALAS2* | *MYLIP* |  |
|  | *POLE2* |  |
|  | *SELENBP1* |  |
|  | *SREBF1* |  |

**Table S3.** Gene expression associated with statin use (Stat), cg06500161 methylation (CpG) and fasting insulin (Ins) in non-diabetic participants from the FHS study (lipids-adjusted model).

| Gene | Phenotype | Chr: Position* | N | β | SE | *P-* value |
| --- | --- | --- | --- | --- | --- | --- |
| *SELENBP1* | Stat | chr1:151364302 | 1616 | 0.0777 | 0.0318 | 0.0144 |
|  | CpG | chr1:151364302 | 1604 | 7.43E-3 | 3.06E-3 | 0.0153 |
|  | Ins | chr1:151364302 | 1616 | 0.0797 | 0.0233 | 6.16E-4 |
| *TTLL4* | Stat | chr2: 218710835 | 1616 | 0.226 | 0.0981 | 0.0212 |
|  | CpG | chr2: 218710835 | 1604 | 0.0231 | 8.38E-3 | 5.78E-3 |
|  | Ins | chr2: 218710835 | 1616 | 0.142 | 0.0566 | 0.0120 |
| *ACSL3* | Stat | chr2:222860934 | 1616 | -0.175 | 0.0700 | 0.0125 |
|  | CpG | chr2:222860934 | 1604 | -0.0191 | 6.04E-3 | 1.53E-3 |
|  | Ins | chr2:222860934 | 1616 | -0.0996 | 7.64E-3 | 0.0161 |
| *KAT2B* | Stat | chr3:20040023 | 1616 | 0.0900 | 0.0439 | 0.0403 |
|  | CpG | chr3:20040023 | 1604 | 8.25E-3 | 3.95E-3 | 0.0366 |
|  | Ins | chr3:20040023 | 1616 | 0.0594 | 0.0277 | 0.0322 |
| *BST1* | Stat | chr4:15702950 | 1616 | 0.140 | 0.0596 | 0.0191 |
|  | CpG | chr4:15702950 | 1604 | 0.0127 | 4.94E-3 | 9.82E-3 |
|  | Ins | chr4:15702950 | 1616 | 0.0894 | 0.0371 | 0.0160 |
| *MEF2C* | Stat | chr5: 88717117 | 1616 | -0.143 | 0.0602 | 0.0175 |
|  | CpG | chr5: 88717117 | 1604 | -0.0130 | 5.29E-3 | 0.0143 |
|  | Ins | chr5: 88717117 | 1616 | -0.121 | 0.0394 | 2.05E-3 |
| *AKAP12* | Stat | chr6:151239815 | 1616 | -0.133 | 0.0597 | 0.0258 |
|  | CpG | chr6:151239815 | 1604 | -0.0155 | 5.17E-3 | 2.64E-3 |
|  | Ins | chr6:151239815 | 1616 | -0.111 | 0.0347 | 1.31E-3 |
| *IGF2R* | Stat | chr6:159969082 | 1616 | 0.161 | 0.0737 | 0.0290 |
|  | CpG | chr6:159969082 | 1604 | 0.0141 | 6.22E-3 | 0.0236 |
|  | Ins | chr6:159969082 | 1616 | -0.125 | 0.0522 | 0.0162 |
| *MYLIP* | Stat | chr6:16129125 | 1616 | -0.364 | 0.0588 | 6.33E-10 |
|  | CpG | chr6:16129125 | 1604 | -0.0243 | 5.07E-3 | 1.67E-6 |
|  | Ins | chr6:16129125 | 1616 | -0.190 | 0.0345 | 3.95E-8 |
| *SQLE* | Stat | chr8:124998506 | 1616 | 0.230 | 0.0530 | 1.47E-5 |
|  | CpG | chr8:124998506 | 1604 | 8.93E-3 | 4.52E-3 | 0.0482 |
|  | Ins | chr8:124998506 | 1616 | 0.0974 | 0.0302 | 1.28E-3 |
| *ABCA1* | Stat | chr9:104781002 | 1616 | -0.262 | 0.0353 | 1.21E-13 |
|  | CpG | chr9:104781002 | 1604 | -0.0152 | 2.97E-3 | 3.48E-7 |
|  | Ins | chr9:104781002 | 1616 | -0.104 | 0.0199 | 1.43E-7 |
| *ST6GALNAC4* | Stat | chr9:127907886 | 1616 | 0.197 | 0.0840 | 0.0191 |
|  | CpG | chr9:127907886 | 1604 | 0.0163 | 7.41E-3 | 0.0275 |
|  | Ins | chr9:127907886 | 1616 | 0.107 | 0.0509 | 0.0352 |
| *MICAL2* | Stat | chr11:12138388 | 1616 | 0.153 | 0.0538 | 4.47E-3 |
|  | CpG | chr11:12138388 | 1604 | 0.0167 | 5.17E-3 | 1.22E-3 |
|  | Ins | chr11:12138388 | 1616 | 0.0903 | 0.0389 | 0.0201 |
| *CINP* | Stat | chr14:102348282 | 1616 | -0.144 | 0.0502 | 4.01E-3 |
|  | CpG | chr14:102348282 | 1604 | -0.0111 | 4.24E-3 | 8.95E-3 |
|  | Ins | chr14:102348282 | 1616 | 0.0629 | 0.0288 | 0.0293 |
| *ABCG1* | Stat | chr21:42219118 | 1616 | -0.599 | 0.0881 | 1.07E-11 |
|  | CpG | chr21:42219118 | 1604 | -0.0636 | 7.33E-3 | 4.09E-18 |
|  | Ins | chr21:42219118 | 1616 | -0.300 | 0.0495 | 1.36E-9 |
| *ALAS2* | Stat | chrX:55009055 | 1616 | 0.0490 | 0.0249 | 0.0491 |
|  | CpG | chrX:55009055 | 1604 | 4.33E-3 | 2.13E-3 | 0.0425 |
|  | Ins | chrX:55009055 | 1616 | 0.0595 | 0.0152 | 8.92E-5 |

* The physical positions of CpG sites are based on GRCh38.p12.

**Table S4.** Gene expression associated with statin use (Stat), cg06500161 methylation (CpG) and fasting glucose level (Glu) in non-diabetic participants from the FHS study (lipids-adjusted model).

| Gene | Phenotype | Chr: Position* | N | β | SE | *P-* value |
| --- | --- | --- | --- | --- | --- | --- |
| *SELENBP1* | Stat | chr1:151364302 | 1616 | 0.0777 | 0.0318 | 0.0144 |
|  | CpG | chr1:151364302 | 1604 | 7.43E-3 | 3.06E-3 | 0.0153 |
|  | Glu | chr1:151364302 | 1616 | 0.0108 | 4.34E-3 | 0.0127 |
| *ACSL3* | Stat | chr2:222860934 | 1616 | -0.175 | 0.0700 | 0.0125 |
|  | CpG | chr2:222860934 | 1604 | -0.0191 | 6.04E-03 | 1.53E-3 |
|  | Glu | chr2:222860934 | 1616 | -0.0195 | 8.06E-3 | 0.0157 |
| *MEF2C* | Stat | chr5:88718241 | 1616 | -0.143 | 0.0602 | 0.0175 |
|  | CpG | chr5:88718241 | 1604 | -0.0129 | 5.29E-3 | 0.0143 |
|  | Glu | chr5:88718241 | 1616 | -0.0302 | 7.50E-3 | 5.69E-5 |
| *ABCA1* | Stat | chr9:104781002 | 1616 | -0.262 | 0.0353 | 1.21E-13 |
|  | CpG | chr9:104781002 | 1604 | -0.0152 | 2.97E-3 | 3.48E-7 |
|  | Glu | chr9:104781002 | 1616 | -8.42E-3 | 3.86E-3 | 0.0291 |
| *SPTB* | Stat | chr14:64746283 | 1616 | 0.154 | 0.0382 | 5.67E-5 |
|  | CpG | chr14:64746283 | 1604 | 9.61E-3 | 3.62E-3 | 7.87E-3 |
|  | Glu | chr14:64746283 | 1616 | 0.0140 | 5.09E-3 | 5.79E-3 |
| *ABCG1* | Stat | chr21:42219118 | 1616 | -0.599 | 0.0881 | 1.07E-11 |
|  | CpG | chr21:42219118 | 1604 | -0.0636 | 7.33E-3 | 4.09E-18 |
|  | Glu | chr21:42219118 | 1616 | -0.0232 | 9.61E-3 | 0.0159 |
| *IL17REL* | Stat | chr22:49994513 | 1616 | -0.254 | 0.113 | 0.0250 |
|  | CpG | chr22:49994513 | 1604 | -0.0247 | 9.47E-3 | 9.01E-3 |
|  | Glu | chr22:49994513 | 1616 | -0.0269 | 0.0126 | 0.0324 |

* The physical positions of CpG sites are based on GRCh38.p12.

**E**

**beta 4.1879**

**SE 0.6026**

**95% CI [3.5853, 4.7906]**

***P* value 3.0298E-42**
